# NIR-Based Adulteration Screening of *Rubus chingii* Hu: A Two-Dimensional Correlation Spectroscopy-Guided Chemometric Strategy with Model-Dependent Variable Selection

**DOI:** 10.3390/foods15142488

**Published:** 2026-07-14

**Authors:** Yu Wang, Yuting Wang, Yi Chen

**Affiliations:** 1 School of Food Science, Nanchang University, Qingshan Lake Campus, No. 235 Nanjing East Road, Nanchang 330047, China; wyu9954@163.com (Y.W.); wangyuting@ncu.edu.cn (Y.W.); 2 State Key Laboratory of Food Science and Resources, Qingshan Lake Campus, No. 235 Nanjing East Road, Nanchang 330047, China

**Keywords:** *Rubus chingii* Hu, near-infrared spectroscopy, adulteration, two-dimensional correlation spectroscopy, wavenumber selection

## Abstract

Economically motivated adulteration (EMA) of high-value geographical indication (GI) food ingredients poses serious risks to consumer trust and food safety. *Rubus chingii* Hu (RcH), a GI-designated edible-medicinal fruit originating from Dexing, is susceptible to adulteration with cheaper non-GI sources. This study developed a rapid, non-destructive near-infrared (NIR) spectroscopy approach combined with chemometric modeling for adulteration identification and ratio prediction of GI RcH. Two-dimensional correlation spectroscopy (2D-COS) using adulteration ratio as an external perturbation identified the 4200–6000 cm^−1^ region as the most sensitive spectral window, mainly reflecting changes in proteins, lipids, and carbohydrates. Using this 2D-COS-guided interval, we systematically compared four discriminant models (PLS-DA, SVM-DA, RF-DA, BPNN-DA) under ten preprocessing strategies. SNV + Smo-BPNN-DA achieved 100% test accuracy, while 1st D-RF-DA also showed excellent generalization (A_tes_ = 99.67%). For adulteration ratio prediction, wavenumber selection (CARS, IWOA) significantly improved Raw-SVR performance (R^2^ from 59.8% to 78.6%) but provided no benefit—and sometimes degraded accuracy—for PLS, RF, and BPNN models, revealing a model-dependent effect rarely reported in food adulteration studies. The optimal regression model was SNV-BPNN-R (R^2^ = 96.96%); for computationally constrained scenarios, CARS + IWOA-2nd D-RF-R is recommended (R^2^ ≈ 95.93% with only 20–40 variables). Our findings provide a practical, physically interpretable, and transferable strategy for rapid EMA screening of powder-based GI food products, supporting on-site or online food authenticity control.

## 1. Introduction

*Rubus chingii* Hu (RcH) is a traditional crop widely consumed as both a food and a medicinal herb in China. Among its production regions, Dexing RcH (Jiangxi Province) h as been granted a Geographical Indication (GI) status (AGI00551) due to its superior quality, particularly its high content of bioactive compounds such as rubusoside F5 [1]. GI products typically command premium market prices, which unfortunately creates strong economic incentives for fraudulent practices—most notably, the adulteration of high-value Dexing RcH with cheaper RcH sourced from non-GI regions. Such economically motivated adulteration (EMA) not only undermines consumer trust and fair trade but also poses potential food safety risks, as adulterated batches may introduce unexpected contaminants or allergens, and the lack of traceability hinders effective recall and risk management [2,3]. Therefore, the development of rapid, reliable, and non-destructive methods to authenticate GI plant-derived food ingredients and quantify adulteration levels is of critical importance for food control and regulatory compliance.

Conventional approaches for botanical origin identification and adulteration detection included morphological examination, DNA barcoding, and chromatography-based chemical profiling. However, these methods suffer from several drawbacks: morphological analysis is highly subjective and requires extensive expertise; DNA-based techniques are time-consuming, costly, and cannot differentiate between closely related geographical origins; chromatographic methods involve laborious sample preparation and are unsuitable for high-throughput screening [4]. Consequently, there is an urgent need for a cost-effective, non-destructive, and field-deployable technique that can be used for on-site or online screening of food adulteration. Near-infrared (NIR) spectroscopy has emerged as a powerful analytical tool for food quality and safety assessment due to its advantages of rapid analysis (seconds per sample), no sample pretreatment, non-destructive nature, and the availability of portable instruments [5,6]. It has been successfully applied for adulteration detection in various food matrices, including edible oils, dairy products, honey, and powdered spices [7]. More recently, its potential has been extended to plant-derived food ingredients with dual medicinal–culinary uses, such as *Dendrobium officinale* [8], *Lycium ruthenicum Murr.* [9], and *Panax ginseng* [10]. However, since raw NIR spectra are susceptible to influences from the sample itself and the surrounding environment and contain a large number of highly collinear variables along with physical interferences (e.g., scattering, baseline drift, noise), which must be removed through spectral preprocessing before reliable chemometric modeling [11,12]. And NIR spectra are characterized by their large volume and high dimensionality—wavenumber selection are required before model construction.

Two-dimensional correlation spectroscopy (2D-COS) is a powerful technique that amplifies subtle spectral differences by applying an external perturbation (e.g., adulteration concentration). It not only resolves overlapping peaks but also identifies the specific wavenumber regions most sensitive to the perturbation, thereby providing a physically meaningful basis for selecting analytical spectral windows [13,14]. In the context of food adulteration, 2D-COS can reveal which chemical constituents (e.g., proteins, carbohydrates, lipids) are primarily altered by the addition of adulterants [15,16]. Furthermore, the choice of spectral preprocessing (e.g., standard normal variate, SNV; smoothing; derivatives) can eliminate physical interferences, noise, and baseline drift, thereby ensuring that the spectral signals accurately reflect the underlying chemical information [11,12]. Moreover, wavelength selection strategies can be employed to address issues of high-dimensional collinearity and overfitting by selecting key variables, thereby enhancing both the robustness and predictive accuracy of the model. For instance, competitive adaptive reweighted sampling (CARS) identifies key variables through an iterative selection process based on regression coefficients; and distinguished by its computational efficiency and interpretable results, it was widely applied in the field of spectral analysis [17,18]; improved whale optimization algorithm (IWOA), on the other hand, performed a global search by simulating the foraging behavior of humpback whales; it was highly flexible and particularly well-suited for nonlinear and complex optimization problems [19]. CARS excels at rapidly eliminating redundant variables but is susceptible to the limitations of linear assumptions; IWOA possessed powerful global optimization capabilities, albeit at a high computational cost. Notably, the two exhibit a natural complementarity: CARS can serve as a pre-filter to compress the search space, followed by fine-tuning via IWOA; alternatively, the variable subsets selected by both methods can be combined to enhance model performance. While these techniques can improve model robustness and computational speed for certain algorithms, they may not be universally beneficial for all types of models [20]. A systematic evaluation of how different models respond to preprocessing and wavelength selection is therefore essential for developing practical food adulteration detection systems.

To date, this study aims to: (1) use 2D-COS to identify the adulteration-sensitive wavenumber region of RcH; (2) compare the performance of four discriminant models under ten preprocessing schemes, selecting the optimal combination for distinguishing Dexing RcH, adulterated RcH, and non-Dexing RcH; (3) evaluate the impact of CARS, IWOA, and CARS + IWOA wavelength selection on four regression models: partial least squares regression (PLS-R), support vector regression (SVR), random forest regression (RF-R), backpropagation neural network regression (BPNN-R) for predicting adulteration ratios; and (4) propose model selection guidelines based on the trade-off between accuracy and computational speed. The findings will provide a methodological reference for the rapid, non-destructive, and on-site screening of EMA in GI plant-based food products, thereby contributing to enhanced food authenticity control.

## 2. Materials and Methods

### 2.1. Sample Collection and Preparation of Adulterated Samples

RcH were collected in 2022–2023. Following identification by the manager of the Good Agricultural Practices (GAP) cultivation base at Tianzhihai Pharmaceutical Co., Ltd., Dexing city, China, all samples were confirmed to be RcH. Seven batches of Dexing RcH samples and six batches of non-Dexing RcH samples were randomly selected for the adulteration study. All raw RcH materials were dried at 50 °C for 12 h to ensure low and uniform moisture content. Preparation of samples involving the adulteration of Dexing RcH with non-Dexing RcH was conducted as follows: each Dexing sample batch was individually adulterated with the six non-Dexing sample batches at specific mass ratios, resulting in the preparation of 42 adulterated samples for each adulteration level. The adulteration ratios were set at 5%, 10%, 15%, 20%, 25%, 30%, 40%, 60%, and 80%, yielding a total of 378 adulterated samples. To ensure model stability, within the model simulating the adulteration of Dexing RcH with non-Dexing RcH, spectral data were acquired 10 times for each batch of pure RcH material, resulting in a total of 130 spectral samples. The total number of samples amounted to 508.

### 2.2. Spectral Acquisition

After the collected RcH samples were pulverized and passed through an 80-mesh sieve, NIR spectra were acquired using an integrating sphere diffuse reflectance module equipped with fiber optics using a commercial Infrared spectrometer (Thermo Scientific Nicolet IS50 FT-NIR spectrometer, Waltham, MA, USA). The acquisition parameters were set as follows: spectral range of 4200–12,000 cm^−1^, 32 scans, and a resolution of 4 cm^−1^.

### 2.3. 2D-COS

2D-COS was proposed by Noda et al., which enhanced spectral resolution by extending the original spectrum into a second dimension, thereby enriching spectral information and facilitating the detection of subtle spectral changes [21,22]. Currently, 2D-COS has been applied to the quantitative detection of adulteration in products such as white tea [23] and camellia oil [24]. In this section, 2D-COS performs a correlation analysis on NIR spectra across various concentration gradients—utilizing the adulteration ratio as an external perturbation—to generate synchronous and asynchronous spectra. The intensity of the autocorrelation peaks located along the diagonal of the synchronous spectrum directly quantifies the sensitivity of each specific wavenumber to the adulteration; by extracting local maxima, the characteristic wavelengths most significantly affected by the adulteration can be automatically identified. Conversely, the asynchronous spectrum reveals the sequential order of changes occurring at different wavenumbers, thereby aiding in the elucidation of the interactions between the adulterant components and the matrix. When applied to NIR data comprising 9 different adulteration ratios and 42 replicate measurements, this method effectively amplifies subtle spectral differences and resolves overlapping peaks. Furthermore, it enables the selection of key spectral features without requiring a priori knowledge, thereby providing reliable inputs for subsequent quantitative modeling; consequently, this approach possesses clear physical significance and demonstrates excellent practical feasibility. The synchronous 2D-COS was implemented using MATLAB R2022a (The MathWorks Inc., Natick, MA, USA).

### 2.4. Spectroscopic Preprocessing

A variety of preprocessing techniques were employed, including standard normal variable (SNV), smoothing (Smo), first-derivative (1st D) and second-derivative (2nd D) processing based on the Savitzky–Golay algorithm, including a window width of 5 points and a polynomial order of 2 (quadratic polynomial fitting), as well as combinations of these techniques: SNV + Smo, Smo + 1st D, Smo + 2nd D, SNV + Smo + 1st D, and SNV + Smo + 2nd D. SNV correction helps mitigate scattering effects within the spectral matrix, eliminate physical interferences, improve linearity, and enhance both the interpretability and predictive accuracy of the model [25]. By calculating the local slopes of the spectral curves, 1st D and 2nd D processing effectively correct for baseline drift [26]. Smoothing is utilized to suppress random noise and improve the signal-to-noise ratio, thereby providing a more reliable foundation for subsequent quantitative or qualitative modeling [27]. All preprocessing procedures were also performed using MATLAB R2022a. The preprocessing results are shown in Figure S1.

### 2.5. Model Building

Before model construction, the ‘evalclusters’ and ‘iforest’ functions of the “Statistic and Machine Learning Toolbox” of MATLAB were utilized to identify spectral outliers among the adulterated samples—based on the degree of separation between sample clusters—thereby enhancing the stability and accuracy of the subsequent classification and regression prediction models. It was reported that the processes of variable selection and outlier detection typically influence one another, and the order in which they were executed could have a significant impact on the final modeling results [28,29]. In the dataset involving the adulteration of Dexing RcH with non-Dexing RcH, a total of six outliers were removed, comprising four Dexing RcH samples, one adulterated sample, and one non-Dexing RcH sample.

#### 2.5.1. Discriminant Models

(1) Dataset Partitioning

A stratified random sampling method was employed to partition the dataset into training and testing sets. Based on the 11 continuous values of the adulteration ratio, samples within each concentration group were randomly divided into training and testing sets at a ratio of 70% to 30%, ensuring that at least one test sample was retained in each group. Following a workflow of wavenumber selection followed by model construction, discriminant models and adulteration prediction models were established separately for PLS, SVM, RF, and BPNN. The sampling process was independently repeated 20 times to mitigate the impact of sampling randomness on model evaluation. The final output parameters of the model consist of the mean plus the standard deviation derived from 20 iterations. The discrimination models and regression models were performed by MATLAB R2022a using self-coded code.

(2) Discriminant Models
  (a) PLS-DA Model: Class labels were converted into dummy variables. Subsequently, a PLS-DA model was constructed using a predetermined number of latent variables (LVs). The classification accuracy of the model was then calculated for both the training and test sets.
  (b) SVM-DA Model: Labels were converted into dummy variables. A grid search combined with cross-validation was employed to optimize the penalty factor C (ranging from 0.1, 1, 10, to 100) and the kernel parameter gamma (ranging from 0.001, 0.01, 0.1, to 1) for the radial basis function (RBF) kernel. Using the optimal parameter combination, a model was established based on a “one-against-all” strategy, followed by the generation of prediction results for the training and test sets.
  (c) RF-DA Model: The data underwent standardization, and labels were converted into dummy variables. A grid search was performed across various combinations of the number of decision trees (50, 100, 200, 500) and the minimum leaf node size (1, 5, 10, 20). Utilizing these optimal parameters, a Random Forest classification model was constructed to predict the class labels for the training and test sets.
  (d) BPNN-DA Model: The data were normalized to the interval (−1, 1), and labels were converted into dummy variables. A single-hidden-layer feed-forward neural network was employed, with the number of hidden-layer nodes set to 7. The selected training function was the Levenberg–Marquardt algorithm (trainlm), with the maximum number of iterations set to 200 and the target error threshold set to 10^−5^. Predictions were generated separately for the training and test sets.

The evaluation metrics utilized were the accuracy on the training set (A_tra)_, accuracy on the testing set (A_tes_), model correlation coefficient on the training set (R_tra_), and testing set (R_tes_).

#### 2.5.2. Regression Models

**Figure 1:**
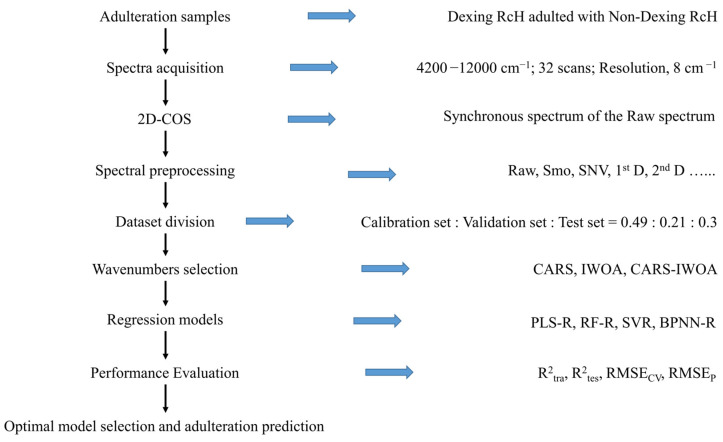
Methodological flowchart of implementation of regression models.

The flowchart of implementation of regression models were shown in Figure 1, and the specific process was as follows:

(1) Dataset Partitioning

All samples were divided into a training set and testing set at a ratio of 7:3. Specifically, the training set was furtherly divided into the calibration and validation set at a ratio of 7:3. Stratified random sampling was employed to partition the samples, ensuring the objectivity of the chemical composition distribution within each set. Notably, the external validation samples were completely excluded from any model training or parameter optimization procedures and were only used to evaluate the final predictive performance after model determination.

(2) Wavenumber selection
  (a) CARS wavenumber selection

First, the optimal number of principal components for PLS regression is determined based on Monte Carlo cross-validation (MCCV): the maximum number of principal components is set to 15, and 50 MCCV runs are performed, with 70% of the samples randomly selected as the training set in each run; the optimal number of principal components is determined based on the criterion of minimizing the cross-validation root mean square error (RMSE). Subsequently, the CARS algorithm is executed using this optimal number of principal components as a parameter, employing 5-fold cross-validation over 100 iterations; through an exponential decay function and adaptive reweighted sampling, unimportant variables are progressively eliminated, ultimately yielding the selected feature subset.

  (b) IWOA wavenumber selection

The IWOA is utilized for spectral variable selection. The algorithm’s population size is set to 25, with a maximum of 50 iterations, and the search space consists of binary vectors (where 0 indicates exclusion and 1 indicates selection). The fitness function is defined as RMSE of the 5-fold CV based on PLS regression, wherein an inner 3-fold cross-validation loop is used to automatically determine the number of PLS principal components (with a maximum limit of 15). During the iterative process, IWOA incorporates quasi-opposition-based learning initialization, a nonlinear convergence factor, adaptive weights, and a stochastic differential mutation strategy to balance global exploration with local exploitation capabilities, ultimately outputting the binary feature mask that minimizes the fitness value.

  (c) CARS-IWOA Cascaded wavenumber selection

A cascaded strategy is adopted to achieve efficient dimensionality reduction: first, CARS is utilized to rapidly screen the original full-spectrum variables, identifying a subset of variables that exhibit strong correlations with the dependent variable while eliminating a large number of redundant and noisy variables; subsequently, this subset serves as the initial search space for IWOA, which then further refines the selection to identify the optimal feature combination. This strategy effectively leverages both the high-efficiency screening capability of CARS and the global optimization capability of IWOA, thereby significantly reducing computational overhead while ensuring effective feature selection performance.

(3) Models construction
  (a) PLS-R

PLS-R is employed to establish a linear predictive model between selected wavenumbers and concentration levels. On the training set, 5-fold stratified CV (5-FS_CV_) is utilized to determine the optimal number of LVs, using the minimization of the RMSE_CV_ as the selection criterion. Subsequently, the final PLS model is constructed using the entire training set and this optimal number of latent variables, and is then applied to make predictions on the test set.

  (b) SVR

SVR employs a radial basis function (RBF) kernel. The grid search range for hyperparameters is defined as follows: penalty coefficient C (0.1, 1, 10, 100) and kernel parameter γ (0.1, 1, 10, 100). On the training set, 5-FS_CV_ is used to evaluate the generalization performance of each set of hyperparameters, with the combination yielding the minimum cross-validation RMSE selected as the optimal set. Finally, an SVR model is built based on the entire training set and the optimal hyperparameters to predict the test set.

  (c) RF-R

RF-R adopts a Bagging ensemble strategy. The candidate hyperparameters include: the number of decision trees (50, 100, 200) and the minimum number of samples per leaf node (1, 5, 10). The optimal combination of hyperparameters is selected via 5-FS_CV_. The final model is constructed using the entire training set and the optimal parameters and is then used to predict the test set.

  (d) BPNN-R

BPNN-R employs a single hidden layer structure. The candidate numbers of hidden layer nodes are (5, 10, 15). Network training is performed using the Levenberg–Marquardt algorithm, with a maximum iteration limit of 200 and a target error of 1 × 10^−5^. To eliminate the influence of data dimensionality, the input variables undergo Z-score standardization. 5-FS_CV_ is used to select the optimal number of hidden layer nodes; the final model is trained using the entire training set and the optimal structure, and is then used to predict the test set.

(4) Model evaluation parameters. The performance of each regression model was evaluated using the following metrics: coefficient of determination of training set (R^2^_tra_) and testing set (R^2^_tes_), RMSE_CV_, and test set prediction root mean square error (RMSE_P_). To avoid overfitting and optimize model parameters, 5-FS_CV_ was applied to the training set, with RMSE_CV_ calculated as the average over the 5 folds. Prior to modeling, all spectral data were mean-centered and scaled to unit variance. To ensure statistical robustness, the entire modeling procedure—including sample splitting, cross-validation, and model training—was repeated 20 times with independent random seeds, and the final results are reported as the mean ± standard deviation of each metric across these 20 iterations.

## 3. Results

### 3.1. Spectral Analysis and 2D-COS

**Figure 2:**
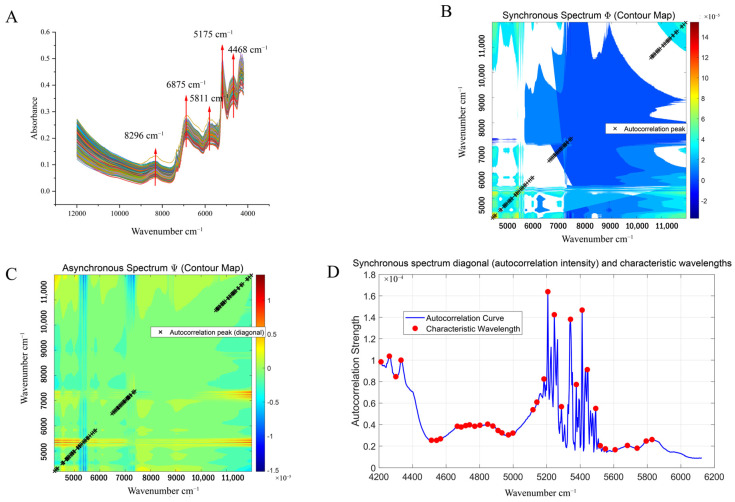
The NIR spectra and 2D-COS analysis of 378 adulterant RcH samples. (**A**) The NIR spectra; (**B**) Synchronous spectrum; (**C**) Asynchronous spectrum; (**D**) Characteristic wavenumber autocorrelation intensity in selected wavenumber range of 4200–6000 cm^−1^.

Figure 2A shows the overlaid raw NIR spectra of all 378 samples. As illustrated, all samples exhibited generally similar spectral profiles, with systematic differences in absorption intensities corresponding to varying adulteration levels. The observed absorption signals arose from overtones and combination bands associated with O-H and C-H bond vibrations; characteristic peak assignments were determined by correlating these signals with matrix components such as moisture, sugars, polyphenols, and cellulose. The peak at 8296 cm^−1^ was assigned to the second overtone of saturated alkyl C-H bonds—corresponding to the alkyl backbones of polysaccharides and cellulose—and served as an indicator for exogenous adulterants like plant fibers and starches. The peak at 6875 cm^−1^ corresponded to the first overtone of O-H bonds, reflecting the characteristic response of both free water and hydrogen-bonded hydroxyl groups, making it suitable for detecting adulteration involving water injection to increase weight. The peak at 5811 cm^−1^ originated from the overtone and combination bands of aromatic and aliphatic C-H bonds; it corresponded to the aromatic structures of polyphenols (such as tannins and flavonoids) in RcH, allowing for the differentiation of adulterants with low polyphenol content. The peak at 5175 cm^−1^ was attributed to the combination band of O-H stretching and water molecule bending; it was highly sensitive to changes in soluble sugars and bound water, enabling the detection of adulteration involving added sugars. The peak at 4668 cm^−1^ resulted from the combination band of C-H and C-O bending vibrations; it served as a specific fingerprint for carbohydrates, characterizing both endogenous and exogenously added sugars within the samples. Absorbance values varied in a gradient corresponding to the level of adulteration, as the adulterants altered the total content of functional groups associated with moisture, carbohydrates, polyphenols, and fibers. Consequently, these characteristic wavenumbers can serve as key variables for qualitative and quantitative modeling to detect RcH adulteration.

2D-COS were generated using the adulteration ratio (0%, 5%, 10%, 15%, 20%, 25%, 30%, 40%, 60%, 80%, and 100%) as an external perturbation. The synchronous spectrum (Figure 2B) revealed that the spectral region most significantly affected by adulteration was 4200–6000 cm^−1^, along with minor contributions from 6500 to 7300 cm^−1^ and 11,400–11,900 cm^−1^. The auto-correlation peaks located on the diagonal of the synchronous spectrum quantitatively reflect the sensitivity of each wavenumber to the adulteration level. Within the 4200–6000 cm^−1^ window, the ten highest auto-correlation intensities were observed at 5206.86, 5411.28, 5245.43, 5341.85, 4261.91, 4331.34, 4211.77, 5442.13, 4300.48, and 5183.72 cm^−1^ (in descending order, Table 1). These wavenumbers correspond to O–H, C–H, and N–H combination bands, indicating that adulteration induces variations in moisture, carbohydrates, lipids, and proteins.

The asynchronous spectrum (Figure 2C) provided additional information on the sequential order of spectral changes. For a representative pair of wavenumbers, 4512.61 cm^−1^ (protein-related N–H combination) and 4211.77 cm^−1^ (lipid/protein C–H + C=O), the synchronous cross-peak was positive (Φ = 4.45 × 10^−5^) and the asynchronous cross-peak was negative (Ψ = −2.89 × 10^−6^). According to Noda’s rules, this combination (Φ > 0, Ψ < 0) indicates that the change at 4512.61 cm^−1^ occurs earlier than that at 4211.77 cm^−1^. Thus, protein-related spectral variations precede lipid-related variations during the adulteration process [30].

### 3.2. The Results of Discrimination Models

**Figure 3:**
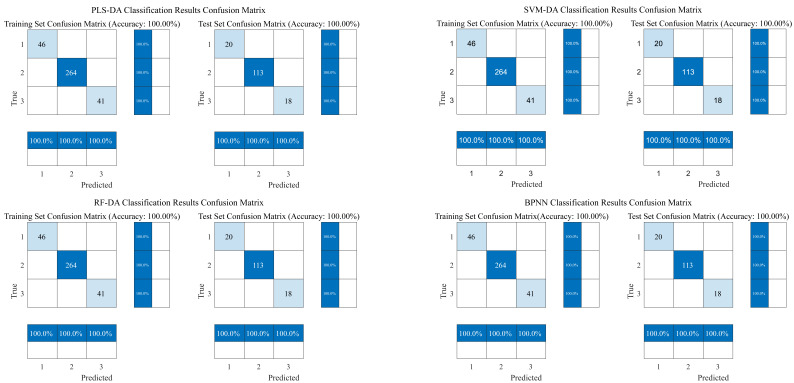
The best model among the four classification models (Abbreviations: PLS-DA, Partial least squares discriminant analysis; SVM-DA, Support vector machine discriminant analysis; RF-DA, Random forest discriminant analysis; BPNN-DA, Backpropagation neural network discriminant analysis).

The detailed results of all classification models were recorded in Table S1, and Table 2 presented the optimal results obtained from four sets of discriminant models based on different spectral preprocessing methods. Across all four model types, the A_tes_ reached 100%, indicating that all four discriminant models are capable of distinguishing between Dexing RcH, adulterated RcH, and non-Dexing RcH; the visualization results further confirm that all four models exhibited a zero misclassification rate (Figure 3). Furthermore, an examination of the discriminant coefficients for each model reveals that the PLS-DA model yielded the lowest coefficient, followed by the SVM-DA model. Based on a comprehensive evaluation, the 1st D-RF-DA and SNV + Smo-BPNN-DA models are identified as the optimal discriminant models.

### 3.3. The Results of Adulteration Ratio Prediction Models

The specific results for PLS-R, SVR, RF-R, and BPNN-R are shown in Tables S2–S5.

#### 3.3.1. PLS-R Models

Under preprocessing methods such as “Raw” and SNV, the R^2^_tes_ ranged from 92.7% to 93.0%, with an RMSE_P_ of approximately 0.09. Following the application of CARS wavenumber selection, R^2^_tes_ declined to around 91.6%; IWOA further reduced it to 87.9%; and the combined CARS-IWOA approach also yielded results inferior to the scenario without wavenumber selection (approximately 90.9%). For 1st D preprocessing, R^2^_tes_ stood at 90.5% without wavenumber selection, but dropped to the range of 87.9–89.1% after wavenumber selection. Under 2nd D preprocessing, the original model performance was already poor (R^2^_tes_ = 78.1%), and wavenumber selection caused it to deteriorate further to the range of 70.8–72.9%. In summary, wavenumber selection exerted no positive effect on the PLS model; on the contrary, it diminished the model’s generalization capability.

#### 3.3.2. SVR Models

In the absence of wavenumber selection, under “Raw” preprocessing, R^2^_tes_ was merely 59.8%, with an RMSE_P_ of 0.21. Following CARS wavenumber selection, R^2^_tes_ improved to 76.1%, and RMSE_P_ decreased to 0.16; IWOA yielded comparable results; and the combined CARS-IWOA approach performed best (R^2^_tes_ = 78.6%, RMSEP = 0.15). For SNV preprocessing, R^2^_tes_ was 38.1% without wavenumber selection, but improved substantially to the range of 84.7–86.1% after wavenumber selection. Under 1st D and 2nd D preprocessing, R^2^_tes_ values were negative in the absence of wavenumber selection (indicating model failure); although the values remained relatively low after wavenumber selection (peaking at 75.2%), they had recovered to a usable range. Wavenumber selection thus demonstrated a significant improvement in the SVR model.

#### 3.3.3. RF-R Models

In the RF Model, when without wavenumber selection, the R^2^_tes_ under “Raw” preprocessing stood at 55.7%; following wavenumber selection, it declined slightly to a range of 50.3–53.1%. Under high-quality preprocessing methods—such as 1st D—the R^2^_tes_ reached 94.1% without wavenumber selection, subsequently dropping to between 91.3% and 93.9% after wavenumber selection. Under second-order derivative preprocessing, the original model yielded an R^2^_tes_ of 93.9%; however, wavenumber selection resulted in a fluctuating decline in performance (reaching a low of 90.9%). Consequently, wavenumber selection offers no benefit to the RF model and may even slightly degrade its performance.

#### 3.3.4. BPNN-R Models

Without wavenumber selection, the R^2^_tes_ under “Raw” preprocessing was 95.0%, with an RMSE_P_ of 0.07. Following wavenumber selection, performance declined significantly: the R^2^_tes_ dropped to 82.4% with CARS, 85.4% with IWOA, and 87.3% with the CARS-IWOA combination. Other preprocessing methods (such as SNV and Smo) exhibited the same pattern, wherein wavenumber selection consistently led to a decrease in R^2^_tes_ by 3 to 10 percentage points. Only a very few specific combinations (e.g., IWOA under Smo + 1st D preprocessing) showed a slight improvement, though these results were accompanied by relatively large standard deviations. Thus, wavenumber selection proves detrimental to the BPNN model.

#### 3.3.5. Optimal Regression Models Under Different Wavenumber Selection Methods

**Figure 4:**
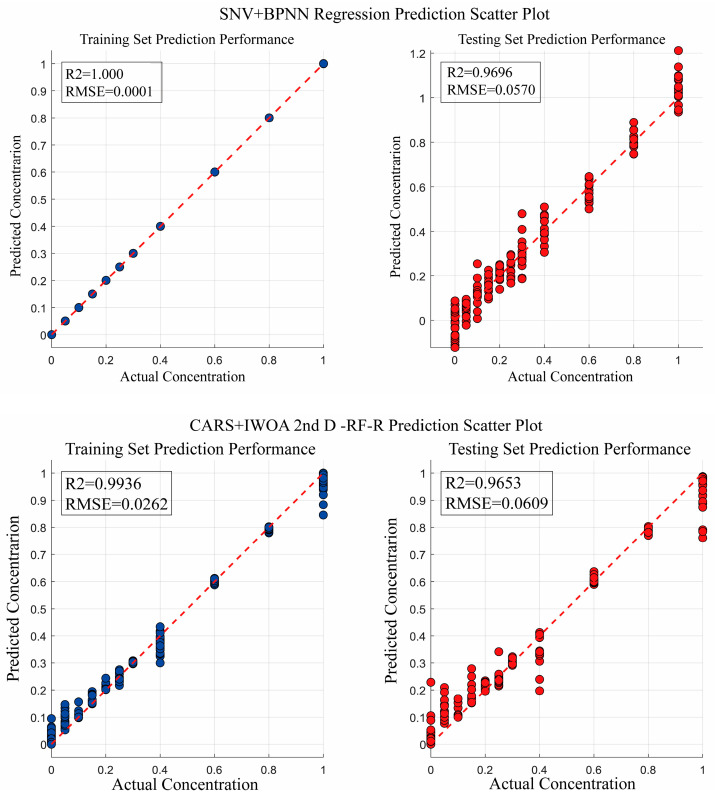
The Sscatter plots of the SNV-BPNN-R and CARS + IWOA-2nd D RF-R regression models. (Abbreviation: SNV-BPNN-R, Backpropagation neural network regression model based on standard normal variate; CARS + IWOA-2nd D RF-R, Random forest regression model based on competitive adaptive reweighted sampling—improved whale optimization algorithm, integrated with second-order derivative).

Table 3 presents the optimal results obtained from four sets of discriminant models. As indicated in the table, the performance of models without wavenumber selection surpasses that of models employing wavenumber selection; however, there are no significant differences in the performance of the optimal regression models constructed using the three distinct wavenumber selection methods. Since the optimal model without wavenumber selection is constructed using a BPNN—which involves the maximum number of input variables—its computational speed is significantly slower than that of the best models obtained through CARS and CARS-IWOA wavenumber selection. In terms of model performance, the SNV-BPNN-R model (without wavenumber selection) stands as the optimal choice; however, if computational speed is the primary consideration, the CARS-IWOA-2nd D RF-R model is the optimal choice. The prediction scatter plots are shown in Figure 4.

## 4. Discussion

The objective of this study was not merely to develop another NIR-based adulteration detection method, but to systematically investigate how three interrelated factors–spectral feature selection (via 2D-COS), preprocessing, and chemometric model choice–affect both discriminant and quantitative performance. Our results revealed a consistent, overarching pattern: the optimal analytical strategy is highly task- and model-dependent. No single preprocessing method or variable selection strategy works universally across all models. In the following discussion, we first demonstrate how 2D-COS provides a physically interpretable spectral window that reduces dimensionality while preserving chemical information. We then compared four discriminant models to show how each responds differently to preprocessing, leading to clear recommendations for authenticity screening. Finally, we examined quantification models, where wavenumber selection proves beneficial only for noise-sensitive models (SVR) but detrimental to others (PLS, RF, BPNN). Collectively, these findings provided a rational, step-by-step workflow that is transferable to other powder-based food authentication tasks and directly applicable to regulatory and industrial settings.

Before any model can be built, the raw spectral data must be reduced to meaningful, interpretable inputs. In this study, 2D-COS served as the critical first step. By using the adulteration ratio as an external perturbation, 2D-COS offered two distinct advantages over conventional one-dimensional difference spectra [31,32]. The synchronous spectrum enhanced spectral resolution by spreading overlapping peaks along the second dimension, allowing unambiguous assignment of adulteration-induced changes to specific wavenumbers; the asynchronous spectrum further revealed the temporal order of chemical events, providing mechanistic insight into how different food components respond to the introduction of adulterants.

From the synchronous spectrum, the 4200–6000 cm^−1^ region emerged as the most sensitive window, with ten characteristic wavenumbers (Table 1) assigned to O–H, C–H, and N–H combination bands. These correspond to moisture, carbohydrates, lipids, and proteins—the primary nutrient categories affected by adulteration. The strong O-H related auto-correlations at 5207 and 5442 cm^−1^ reflect changes in water and cellulosic polysaccharides [33]; the intense C-H peaks at 5411, 5342, 4331 and 4300 cm^−1^ indicate alterations in lipid profiles [34], known to vary with geographical origin and fruit maturity; and the bands at 4212, 4262 and 5184 cm^−1^ point to protein and ester bond variations [35]. Collectively, these assignments demonstrate that adulteration primarily affects lipid and protein fractions, accompanied by secondary changes in carbohydrates and moisture–a finding consistent with known compositional differences between GI and non-GI RcH.

More importantly, the asynchronous spectrum revealed that protein-related changes (e.g., at 4513 cm^−1^) occur earlier than lipid-related changes (e.g., at 4212 cm^−1^). This sequential order suggests a mechanistic hypothesis: upon adulteration, the non-authentic powder first alters the protein matrix (through differences in protein content or secondary structure), which then triggers adjustments in lipid organization. From a food control perspective, this insight has a direct practical implication: for early detection of low-level adulteration (e.g., 5–15%), models should prioritize protein-sensitive wavenumbers (such as 4513 or 5184 cm^−1^) to achieve higher sensitivity before significant lipid changes become evident.

Thus, 2D-COS not only pinpoints a physically meaningful spectral window but also provides a rational, interpretable basis for subsequent chemometric modeling. The selected 4200–6000 cm^−1^ interval reduces input dimensionality from ~2000 to ~520 variables (or even to 10 key wavenumbers), which markedly decreases computational load and facilitates deployment on portable NIR devices for on-site screening. More importantly, because the selected wavenumbers have clear chemical assignments, the resulting models are transparent and interpretable—i.e., their predictions can be traced back to specific chemical constituents. This explainability is particularly critical for regulatory acceptance (as agencies increasingly require scientifically defensible evidence for food authenticity testing) and also bolsters consumer confidence by ensuring that adulteration detection is grounded in verifiable chemistry rather than statistical correlation alone.

After identifying the most information-rich spectral region, spectral preprocessing was applied to this specific area, which was subsequently utilized to construct both classification models and prediction models. The study revealed that the impact of preprocessing on discriminative and predictive performance depends largely on the specific model employed, reflecting the unique mathematical principles inherent to each type of model [12].

Among the classification models constructed using PLS, SVM, RF, and BPNN, the baseline drift, scattering, and noise inherent in the raw spectra exert vastly different effects on the various models. RF-DA suffers from severe overfitting when applied to raw spectra (with a R_tes_ of only 59.16%), as the decision trees repeatedly exploit non-informative spectral variations during the splitting process; conversely, BPNN-DA—leveraging deep nonlinear mapping—achieves a test accuracy of 99.7% even without any preprocessing, thereby demonstrating the neural network’s adaptive capability to suppress inherent spectral interferences [36]. 1st D transformation effectively eliminates additive baselines and enhances the contrast between spectral peaks and valleys, though it simultaneously amplifies high-frequency noise. SVM-DA and RF-DA are highly sensitive to local spectral features and benefit significantly from 1st D preprocessing (achieving A_tes_ of 100% and 99.67%, respectively): for SVM, the kernel hyperplane becomes more readily linearly separable, while for RF, the splitting nodes show a greater propensity to select wavelength regions with genuine chemical significance [37]. Applying smoothing before differentiation effectively mitigates noise amplification; consequently, the performance of the “Smo + 1st D” approach generally surpasses that of differentiation alone. Although the 2nd D transformation can eliminate baseline curvature, it severely amplifies noise and may potentially invert peak positions. PLS-DA proves most sensitive to 2nd D preprocessing, with its test R^2^ dropping from 94.22% to 87.20%; this is because the model relies on the covariance structure among variables, and the amplified noise directly disrupts these underlying linear relationships [38]. RF-DA and BPNN-DA exhibit slightly higher tolerance to second-derivative preprocessing, benefiting from the partial noise-suppression effects provided by ensemble averaging and nonlinear activation functions, respectively. While smoothing effectively reduces random noise, it inevitably blurs spectral details. Smoothing applied in isolation yields the poorest performance in RF-DA (with a R_tes_ of only 56.73%); this is because RF relies on capturing subtle spectral fluctuations to extract discriminant information, and smoothing eliminates these informative variations, thereby rendering the model more susceptible to misguidance by residual low-frequency noise. SNV transformation primarily serves to correct for particle scattering effects. It demonstrates stable performance in both PLS-DA and BPNN-DA but offers only limited improvement for RF-DA, as tree-based models rely more heavily on the local shape of the spectrum rather than on overall amplitude normalization.

When the research objective changes from classification to predicting the precise adulteration rate, the role of wavenumber selection becomes model-dependent, which is quite different from the observations of discriminative models. The benefits of CARS, IWOA, or combinations thereof are highly model-specific—a finding that has rarely been systematically reported in near-infrared spectroscopy-based food adulteration studies. For example, the performance of SVR models has been significantly improved, while PLS, RF, and BPNN models either have not benefited or have even experienced a decline in performance. The fundamental reason for this difference is that the models have different degrees of dependence on variable selection, different ways of utilizing information, and different tolerances for input dimensions [39]; in addition, the computational efficiency gains obtained through variable selection must be carefully weighed against the prediction accuracy. Many previous NIR adulteration studies assume that variable selection is universally beneficial. Our results challenge this assumption by demonstrating model-dependent effects. This insight is particularly valuable for regulatory laboratories that wish to deploy rapid screening methods: the decision to perform wavenumber selection should be guided by the selected regression model, not applied as a default preprocessing step.

The aforementioned results indicated that the impact of wavenumber selection varies significantly across different regression models: the performance of SVR improved substantially, whereas PLS, RF, and BPNN either failed to benefit or experienced a decline in performance. The fundamental reason for this divergence lies in the differing dependencies of each model on variable selection, their distinct approaches to utilizing information, and their varying tolerances for input dimensionality [39]; furthermore, the computational efficiency gains yielded by variable selection must be carefully balanced against predictive accuracy.

The SVR model maps data into a high-dimensional space using kernel functions, and its generalization performance is highly contingent upon the signal-to-noise ratio of the input variables. In raw spectral data, the presence of numerous redundant variables and noise can interfere with the selection of support vectors, leading to severe overfitting or underfitting (in this study, the R^2^ value for the Raw + SVR model was merely 59.8%, with an RMSEP of 0.21). CARS eliminates irrelevant variables through competitive adaptive reweighted sampling, retaining 50–100 key wavelengths; IWOA retains approximately 200 variables; and the combined CARS-IWOA strategy further refines this selection down to 20–40 variables. This reduction in the number of variables not only significantly enhances SVR’s predictive capability (with CARS-IWOA boosting the R^2^ value to 78.6% and lowering the RMSEP to 0.15) but also substantially reduces the computational complexity involved in calculating the kernel matrix. Studies have demonstrated that integrating CARS with SVR effectively identifies features strongly correlated with the target variable, thereby reducing the model’s RMSEP by over 20% [17,39]. Although IWOA retains a larger number of variables, it mitigates the problem of local optima through global optimization; the two-step combined strategy further compresses the variable set while maintaining accuracy, resulting in a significant improvement in computational speed.

The PLS model was based on latent variable dimensionality reduction; by extracting a small number of principal components, it effectively achieves the compression and denoising of the full spectrum. Performing additional wavenumber selection not only fails to provide new information but may actually eliminate variables that are relevant to the target yet carry lower weights, thereby disrupting the covariance structure of the PLS model [40]. In this study, following wavenumber selection, the R^2^tes of the PLS model declined from 93.0% to a range of 87.9–91.6%, while the RMSEP generally increased. Although the number of variables was reduced from the full spectrum (ranging from hundreds to thousands) to between 20 and 200—resulting in some improvement in computational speed—this trade-off, made at the expense of predictive accuracy, proves to be a net loss for the PLS model.

The RF model features a built-in mechanism for assessing feature importance; it demonstrates strong robustness against irrelevant variables and, through the use of random sampling and ensemble learning, is already highly effective at mitigating noise interference. In this study, the RF model achieved an R^2^tes of 94.1% when coupled with first-derivative preprocessing; however, following wavenumber selection, this value actually declined to a range of 91.3–93.9%. The decision tree splitting process inherent to the RF model naturally involves the random selection of variable subsets; therefore, forcibly pre-selecting variables may diminish the model’s diversity and compromise its stability [41]. Furthermore, the RF model exhibits low sensitivity to the number of variables—capable of rapid training even when utilizing the full spectrum—meaning that the computational gains derived from variable compression are, in this context, quite limited.

As a deep nonlinear model, the BPNN model relies on rich input information to achieve its powerful fitting capabilities. Wavenumber selection reduces the number of variables from the full spectrum significantly, down to a range of 20 to 200. While this process markedly decreases the number of nodes in the network’s input layer and shortens training times, the resulting loss of information prevents the neural network from learning the complete mapping relationships, thereby leading to underfitting. In this study, the R^2^tes of the Raw + BPNN model was 95.0%; however, following the application of CARS-IWOA, it dropped to 87.3%—a decline of 7.7 percentage points. A very few specific combinations (such as Smo + 1st D + IWOA) showed slight improvements; however, their relatively large standard deviations indicated that the results were unstable.

Taken together, CARS, IWOA, and their combinations demonstrate significant efficacy in reducing the number of input variables (with CARS-IWOA capable of reducing the count to between 20 and 40). This capability substantially accelerates model computation speeds and holds particular practical value for high-dimensional spectral data. Nevertheless, the applicability of wavenumber selection depends on the inherent characteristics of the model itself: SVR is sensitive to high-dimensional noise and relies heavily on variable selection; PLS and RF possess built-in dimensionality reduction or feature selection capabilities, rendering additional external filtering of marginal benefit or even detrimental; conversely, BPNN relies on information richness, meaning that excessive data compression actually compromises predictive accuracy. Therefore, in practical applications, wavenumber selection strategies should be selected judiciously based on the specific model type, and a reasonable balance must be struck between predictive accuracy and computational efficiency [20]. This insight is particularly valuable for regulatory laboratories that wish to deploy rapid screening methods: the decision to perform wavenumber selection should be guided by the selected regression model, not applied as a default preprocessing step.

In summary, this study provides a rational, step-by-step workflow—from 2D-COS-guided feature extraction to model-aware preprocessing and variable selection—that enhances both the accuracy and the interpretability of NIR-based adulteration detection. The derived guidelines are not limited to *Rubus chingii* but are transferable to other powder-based food authentication tasks, as they derive from fundamental model properties rather than sample-specific characteristics.

## 5. Conclusions

This study established a near-infrared (NIR) workflow to authenticate a high-value geographical indication (GI) food product (*Rubus chingii* Hu) against adulteration with non-GI material. Two-dimensional correlation spectroscopy (2D-COS) identified the 4200–6000 cm^−1^ region as the most sensitive spectral window, with auto-correlation peaks assigned to O–H, C–H, and N–H combinations (proteins, lipids, carbohydrates). Asynchronous spectra further revealed that protein-related changes occur earlier than lipid changes, a mechanistic insight that supports early detection of low-level adulteration (e.g., 5–15%) by prioritizing protein-sensitive wavenumbers (e.g., 4512, 5184 cm^−1^). For discrimination, SNV + Smo-BPNN-DA achieved the highest accuracy (100%, R^2^ = 99.9%), while 1st D-RF-DA offered an interpretable alternative (99.7% accuracy). PLS-DA should avoid second-derivative preprocessing, which severely degrades performance. For quantification, wavenumber selection (CARS/IWOA) benefited only the noise-sensitive SVR model (R^2^ from 59.8% to 78.6%); it provided no advantage and often reduced accuracy for PLS, RF, and BPNN. Thus, SNV-BPNN-R without variable selection gave the highest predictive accuracy (R^2^ = 95.0%) for benchtop use, whereas CARS + IWOA-2nd D-RF-R (R^2^ ≈ 90.9%) with only 20–40 variables offers a practical compromise for portable, on-site screening. Overall, this study provides a model-aware, step-by-step strategy—from 2D-COS-guided feature extraction to preprocessing and variable selection—that is transferable to other powder-based GI food authentication tasks, supporting rapid, interpretable, and regulatory-ready anti-fraud screening.

## Figures and Tables

**Table 1:** Peak assignment of the top 10 characteristic wavenumbers in the range of 4200–6000 cm^−1^.

	Wavenumber (cm^−1^)	Autocorrelation Strength	Major Functional Group Affiliation	Possible Composition
1	5206.86	0.000164	O-H stretching + bending combination frequency	Water, cellulose, pectin, and other carbohydrates
2	5411.28	0.000147	C-H stretching combination frequency (CH_2_/CH_3_)	Fatty acids, glycerides, and other lipids
3	5245.43	0.000142	O-H + C-H combination frequency	Carbohydrates and some lipids
4	5341.85	0.000138	C-H stretching combination frequency	Lipids (mainly), proteins
5	4261.91	0.000104	C-H + C=O combination frequency	Lipid ester bonds, protein amide I bands
6	4331.34	0.000100	C-H stretching + deformation combination frequency	Lipids, carbohydrates
7	4211.77	0.0000985	C-H + C=O combination frequency	Proteins (amide I/II), lipids
8	5442.13	0.0000912	O-H stretching combination frequency	Water, cellulose, starch, and other carbohydrates
9	4300.48	0.0000847	C-H stretching + deformation combination frequency	Lipids, hemicellulose
10	5183.72	0.0000825	N–H stretching + amide II combination frequency	Proteins (amide absorption)

**Table 2:** The Best Output Results from Four Discriminant Models.

Models	Preprocessing	A_tra_ (%)	A_tes_ (%)	R_tra_ (%)	R_tes_ (%)
PLS-DA	SNV	100 ± 0	100 ± 0	96.19 ± 0.32	94.48 ± 0.35
SVM-DA	Smo + 1st D	100 ± 0	100 ± 0	98.02 ± 0.07	96.61 ± 0.38
RF-DA	1st D	99.96 ± 0.1	99.67 ± 0.39	99.86 ± 0.2	99.12 ± 1.02
BPNN-DA	SNV + Smo	100 ± 0	100 ± 0	100 ± 0	99.9 ± 0.12

All abbreviations are defined in the main text.

**Table 3:** The Output Resrults of Optimal Regression Models under Different Wavenumber Selection Methods.

Models	Wavenumber Selection	Preprocessing	RMSE_CV_	RMSE_P_	R^2^_tra_ (%)	R^2^_tes_ (%)
PLS-R	Non	SNV	0.09 ± 0	0.09 ± 0.01	96.43 ± 0.26	92.73 ± 1
	CARS	SNV + Smo	0.09 ± 0	0.09 ± 0.01	94.19 ± 0.44	91.89 ± 0.99
	IWOA	Smo + 1st D	0.1 ± 0	0.1 ± 0	95.55 ± 0.79	90.66 ± 0.9
	CARS-IWOA	SNV + Smo	0.09 ± 0.01	0.1 ± 0.01	93.21 ± 0.99	90.87 ± 1.41
SVR	Non	Raw	0.22 ± 0.01	0.21 ± 0.01	91.96 ± 1.31	59.79 ± 3.82
	CARS	SNV + Smo	0.13 ± 0.01	0.13 ± 0.01	93.97 ± 0.62	84.96 ± 3.47
	IWOA	SNV	0.14 ± 0.03	0.14 ± 0.03	94.01 ± 0.81	81.87 ± 8.95
	CARS-IWOA	SNV + Smo	0.12 ± 0.01	0.12 ± 0.01	94.46 ± 0.53	87.23 ± 2.45
RF-R	Non	1st D	0.09 ± 0	0.08 ± 0.01	98.9 ± 0.09	94.11 ± 2.09
	CARS	2nd D	0.08 ± 0.01	0.07 ± 0.01	99.14 ± 0.21	94.93 ± 1.86
	IWOA	2nd D	0.09 ± 0	0.08 ± 0.01	99.03 ± 0.16	94.55 ± 1.89
	CARS-IWOA	2nd D	0.08 ± 0.01	0.07 ± 0.02	99.1 ± 0.33	94.62 ± 2.52
BPNN-R	Non	SNV	0.06 ± 0	0.06 ± 0.01	100 ± 0	96.99 ± 0.61
	CARS	Smo + 1st D	0.08 ± 0.01	0.09 ± 0.01	100 ± 0	93.05 ± 1.63
	IWOA	Smo + 1st D	0.08 ± 0	0.07 ± 0.01	100 ± 0	94.74 ± 0.88
	CARS-IWOA	Smo + 1st D	0.09 ± 0.01	0.09 ± 0.02	99.99 ± 0	91.54 ± 2.68

All abbreviations are defined in the main text.

## Data Availability

The datasets generated and/or analyzed during the current study are not publicly available due to confidentiality but are available from the corresponding author upon reasonable request.

## Supplementary Materials

The following supporting information can be downloaded at: https://www.mdpi.com/article/10.3390/foods15142488/s1, Table S1: Output Results of Four Discriminant Models; Table S2: Output Results of Four Regression Models without wavenumber selection; Table S3: Output Results of Four Regression Models Based on CARS Wavenumber Selection; Table S4: Output Results of Four Regression Models Based on IWOA Wavenumber Selection; Table S5: Output Results of Four Regression Models Based on CARS-IWOA Wavenumber Selection; Figure S1: NIR spectral preprocessing. A, Raw; B, 1st D; C, 2nd D; D, SNV; E, Smo; F, Smo + 1st D; G, Smo + 2nd D; H, SNV + Smo; I, SNV + Smo+1st D; J, SNV + Smo + 2nd D. (All abbreviations are defined in the main text).
