# Supplementary material for: NIR-Based Adulteration Screening of Rubus chingii Hu: A Two-Dimensional Correlation Spectroscopy-Guided Chemometric Strategy with Model-Dependent Variable Selection
Source: Foods. 2026 Jul 14;15(14):2488. doi: 10.3390/foods15142488 (PMC13408191; doi:10.3390/foods15142488)

Table S1 Output Results of Four Discriminant Models

Models	Pre-processing	A_{tra}	A_{tes}	R_{tra}	R_{tes}
PLS-DA	Raw	100±0	100±0	95.98±0.55	94.22±0.73
	1st D	100±0	99.97±0.14	95.76±0.52	93.25±0.74
	2nd D	100±0	99.6±0.44	93.32±0.95	87.2±1.27
	SNV	100±0	100±0	96.19±0.32	94.48±0.35
	Smo	100±0	100±0	95.75±0.52	94.21±0.73
	Smo+1st D	100±0	100±0	96.5±0.32	94.49±0.53
	Smo+2nd D	100±0	99.74±0.61	93.34±1.27	88.85±1.62
	SNV+Smo	100±0	100±0	95.97±0.25	94.11±0.6
	SNV+Smo+1st D	100±0	100±0	96.2±0.49	94.08±0.6
	SNV+Smo+2nd D	100±0	99.74±0.32	93.66±1.41	87.99±1.22
SVM-DA	Raw	99.46±0.55	96.95±1.33	91.17±2.26	84.01±1.83
	1st D	100±0	100±0	97.91±0.07	95.76±0.33
	2nd D	100±0	99.74±0.39	97.58±0.16	93.53±1.03
	SNV	100±0	99.97±0.14	97.39±0.43	95.34±0.74
	Smo	99.34±0.54	96.72±1.28	90.34±1.43	83.01±1.24
	Smo+1st D	100±0	100±0	98.02±0.07	96.61±0.38
	Smo+2nd D	100±0	99.93±0.2	97.64±0.21	94.39±0.73
	SNV+Smo	100±0	99.93±0.2	97.09±0.63	95.13±0.64
	SNV+Smo+1st D	100±0	100±0	98.01±0.06	96.55±0.39
	SNV+Smo+2nd D	100±0	99.77±0.32	97.77±0.11	94.48±0.53
RF-DA	Raw	99.7±1.3	81.26±2.38	93.76±2.21	59.16±4.19
	1st D	99.96±0.1	99.67±0.39	99.86±0.2	99.12±1.02
	2nd D	100±0	99.4±0.62	99.78±0.11	98.59±1.15
	SNV	99.94±0.19	96.75±1.95	98.99±0.3	92.79±2.83
	Smo	99.4±1.76	80.53±2.18	93.09±3.16	56.73±3.91
	Smo+1st D	99.96±0.14	99.07±0.82	99.59±0.24	97.88±1.31
	Smo+2nd D	99.97±0.09	99.44±0.7	99.8±0.14	98.34±1.67
	SNV+Smo	100±0	97.28±1.86	98.99±0.18	93.08±2.97
	SNV+Smo+1st D	99.93±0.15	99.11±0.76	99.56±0.29	98.12±1.34
	SNV+Smo+2nd D	100±0	99.5±0.55	99.77±0.14	98.65±1.05
BPNN-DA	Raw	100±0	99.7±0.57	100±0	97.87±1.2
	1st D	100±0	99.8±0.37	100±0	99.1±1.37
	2nd D	100±0	98.34±1.37	100±0	95.97±3.05
	SNV	100±0	99.74±0.49	100±0	99.38±0.88
	Smo	100±0	99.6±0.92	100±0	98.25±1.36
	Smo+1st D	100±0	99.9±0.24	100±0	99.63±0.53
	Smo+2nd D	100±0	99.5±0.69	100±0	98.21±1.73
	SNV+Smo	100±0	100±0	100±0	99.9±0.12
	SNV+Smo+1st D	100±0	100±0	100±0	99.83±0.32
	SNV+Smo+2nd D	100±0	99.27±0.72	100±0	98.06±1.49

All abbreviations are defined in the main text.

Table S2 Output Results of Four Regression Models without wavenumber selection

Models	Propressing	$RMSE_{CV}$	$RMSE_P$	R^2_{tra}	R^2_{tes}
PLS-R	Raw	0.09±0	0.09±0.01	95.63±0.52	93.03±0.98
	1st D	0.1±0	0.1±0.01	96.09±1	90.52±0.96
	2nd D	0.15±0	0.15±0.01	91.66±1.54	78.14±2.26
	SNV	0.09±0	0.09±0.01	96.43±0.26	92.73±1
	Smo	0.09±0	0.09±0	95.18±0.25	92.77±0.73
	Smo+1st D	0.1±0	0.09±0.01	95.96±0.64	91.72±0.91
	Smo+2nd D	0.13±0.01	0.13±0.01	93.5±1.98	82.94±1.63
	SNV+Smo	0.09±0	0.09±0.01	95.62±0.27	92.47±0.89
	SNV+Smo+1st D	0.1±0	0.1±0.01	96.01±0.51	91.3±1
	SNV+Smo+2nd D	0.13±0	0.13±0.01	93.94±1.73	84.05±1.73
SVR	Raw	0.22±0.01	0.21±0.01	91.96±1.31	59.79±3.82
	1st D	0.33±0	0.33±0	91.61±0	-0.33±0.11
	2nd D	0.33±0	0.33±0	91.61±0	-0.97±0
	SNV	0.27±0	0.26±0	91.77±0.05	38.1±2.05
	Smo	0.22±0	0.21±0.01	92.01±1.25	58.21±3.17
	Smo+1st D	0.32±0	0.32±0	91.61±0.01	3.51±0.61
	Smo+2nd D	0.33±0	0.33±0	91.61±0	-0.97±0
	SNV+Smo	0.27±0	0.26±0	91.78±0.06	38.5±2.39
	SNV+Smo+1st D	0.32±0	0.32±0	91.59±0.01	2.23±0.4
	SNV+Smo+2nd D	0.33±0	0.33±0	91.61±0	-0.97±0
RF-R	Raw	0.23±0.01	0.22±0.01	89.3±1.99	55.74±5.68
	1st D	0.09±0	0.08±0.01	98.9±0.09	94.11±2.09
	2nd D	0.1±0	0.08±0.01	98.9±0.18	93.9±2.14
	SNV	0.12±0.01	0.1±0.01	97.9±0.26	90.14±2.37
	Smo	0.23±0.01	0.22±0.01	89.48±1.71	54.3±4.08
	Smo+1st D	0.1±0.01	0.09±0.02	98.68±0.19	92.31±2.69
	Smo+2nd D	0.11±0	0.08±0.01	98.78±0.14	93.12±2.16
	SNV+Smo	0.13±0.01	0.11±0.01	97.54±0.47	88.43±2.56
	SNV+Smo+1st D	0.1±0.01	0.09±0.01	98.68±0.4	92.97±2.27
	SNV+Smo+2nd D	0.1±0.01	0.09±0.01	98.8±0.18	92.73±2.26
BPNN-R	Raw	0.08±0.01	0.07±0.01	100±0	95.03±1.54
	1st D	0.09±0	0.08±0.01	100±0	93.35±1.9
	2nd D	0.13±0.01	0.13±0.01	100±0	83.39±3.13
	SNV	0.06±0	0.06±0.01	100±0	96.99±0.61
	Smo	0.09±0.01	0.08±0.01	100±0	93.59±1.97
	Smo+1st D	0.08±0	0.08±0.01	100±0	94.44±1.03
	Smo+2nd D	0.12±0.01	0.11±0.01	100±0	88.4±1.58
	SNV+Smo	0.07±0	0.06±0.01	100±0	96.55±0.77
	SNV+Smo+1st D	0.08±0	0.08±0.01	100±0	94.43±1.57
	SNV+Smo+2nd D	0.12±0.01	0.11±0.01	100±0	88.34±1.99

All abbreviations are defined in the main text.

Table S3 Output Results of Four Regression Models Based on CARS Wavenumber Selection

Models	Propressing	$RMSE_{CV}$	$RMSE_P$	R^2_{tra}	R^2_{tes}
PLS-R	Raw	0.09±0	0.09±0.01	94.24±0.4	91.55±1.11
	1st D	0.09±0	0.11±0.01	95.46±0.54	89.11±1.69
	2nd D	0.14±0.01	0.17±0.01	88.93±1.52	72.9±2.42
	SNV	0.09±0	0.1±0	94.51±0.37	91.47±0.81
	Smo	0.09±0	0.09±0.01	93.95±0.51	91.51±1.37
	Smo+1st D	0.08±0	0.1±0	95.7±0.48	90.76±0.88
	Smo+2nd D	0.13±0.01	0.15±0.01	91.06±1.41	78.47±2.36
	SNV+Smo	0.09±0	0.09±0.01	94.19±0.44	91.89±0.99
	SNV+Smo+1st D	0.08±0	0.1±0.01	95.64±0.52	90.54±1.32
	SNV+Smo+2nd D	0.12±0	0.14±0.01	91.88±1.09	80.33±2.18
SVR	Raw	0.16±0.01	0.16±0.02	93.57±1.26	76.1±5.37
	1st D	0.21±0.03	0.2±0.03	92.48±0.41	60.38±12.18
	2nd D	0.25±0.04	0.24±0.04	92.39±0.6	43.84±18.17
	SNV	0.13±0.01	0.13±0.01	94.09±0.63	84.74±3.14
	Smo	0.16±0.01	0.16±0.02	93.05±2.35	75.13±6.28
	Smo+1st D	0.18±0.02	0.17±0.02	92.93±0.38	73.63±6.66
	Smo+2nd D	0.25±0.04	0.24±0.04	92.29±0.68	44.04±18.36
	SNV+Smo	0.13±0.01	0.13±0.01	93.97±0.62	84.96±3.47
	SNV+Smo+1st D	0.17±0.02	0.16±0.02	93.05±0.47	76.92±6.11
	SNV+Smo+2nd D	0.24±0.04	0.24±0.04	92.39±0.56	46.05±17.04
RF-R	Raw	0.23±0.01	0.23±0.01	87.6±2.7	50.28±5.92
	1st D	0.1±0.01	0.09±0.02	98.55±0.36	91.25±4.12
	2nd D	0.08±0.01	0.07±0.01	99.14±0.21	94.93±1.86
	SNV	0.13±0.01	0.13±0.01	96.73±0.58	83.81±3.21
	Smo	0.23±0.01	0.23±0.01	87.35±2.32	50.36±5.58
	Smo+1st D	0.11±0.01	0.11±0.02	98.16±0.41	89.14±3.1
	Smo+2nd D	0.1±0.01	0.1±0.02	98.49±0.34	90.93±3.25
	SNV+Smo	0.14±0.01	0.14±0.01	96.18±0.43	82.45±2.82
	SNV+Smo+1st D	0.11±0.01	0.11±0.02	97.98±0.41	88.29±4.31
	SNV+Smo+2nd D	0.1±0.01	0.09±0.01	98.74±0.2	92.2±1.93
BPNN-R	Raw	0.13±0.02	0.13±0.04	99.88±0.21	82.43±9.22
	1st D	0.09±0.01	0.1±0.01	100±0	91.14±2.81
	2nd D	0.14±0.01	0.15±0.02	100±0	78.46±5.07
	SNV	0.1±0.01	0.11±0.02	99.95±0.21	89.17±5.17
	Smo	0.12±0.02	0.13±0.05	99.63±0.51	82.27±16.79
	Smo+1st D	0.08±0.01	0.09±0.01	100±0	93.05±1.63
	Smo+2nd D	0.12±0.01	0.14±0.03	100±0	82.18±6.65
	SNV+Smo	0.1±0.01	0.11±0.04	99.89±0.31	88.39±9.74
	SNV+Smo+1st D	0.08±0.01	0.09±0.01	100±0	92.43±1.87
	SNV+Smo+2nd D	0.11±0.01	0.12±0.02	100±0	85.78±3.94

All abbreviations are defined in the main text.

Table S4 Output Results of Four Regression Models Based on IWOA Wavenumber Selection

Models	Propressing	$RMSE_{CV}$	$RMSE_P$	R^2_{tra}	R^2_{tes}
PLS-R	Raw	0.11±0.01	0.11±0.02	90.88±3.33	87.9±3.4
	1st D	0.11±0.01	0.11±0.01	94.34±1.16	88.9±1.17
	2nd D	0.17±0.01	0.17±0.01	85.23±2.75	72.74±3.31
	SNV	0.11±0.01	0.11±0.01	91.62±2.35	88.47±2.62
	Smo	0.11±0.01	0.11±0.02	91.12±2.94	88.61±3.25
	Smo+1st D	0.1±0	0.1±0	95.55±0.79	90.66±0.9
	Smo+2nd D	0.15±0.01	0.15±0.01	89.22±2.32	79.39±2.95
	SNV+Smo	0.1±0.01	0.1±0.01	92.97±2.46	90.18±2.89
	SNV+Smo+1st D	0.1±0.01	0.1±0.01	95.67±0.54	90.24±1.31
	SNV+Smo+2nd D	0.14±0.01	0.14±0.01	89.87±2.77	80.32±2.8
SVR	Raw	0.17±0.01	0.16±0.02	92.54±2.15	75.91±6.96
	1st D	0.31±0.01	0.31±0.01	91.61±0.03	11.67±7.23
	2nd D	0.33±0	0.33±0	91.6±0.01	-0.54±0.36
	SNV	0.14±0.03	0.14±0.03	94.01±0.81	81.87±8.95
	Smo	0.17±0.01	0.16±0.02	92.83±2.47	74.8±5.25
	Smo+1st D	0.29±0.01	0.28±0.02	91.71±0.07	26.96±9.07
	Smo+2nd D	0.33±0	0.33±0	91.59±0.03	0.22±2.04
	SNV+Smo	0.17±0.04	0.15±0.04	93.34±0.91	76.7±11
	SNV+Smo+1st D	0.29±0.02	0.28±0.02	91.68±0.17	26.57±9.85
	SNV+Smo+2nd D	0.33±0	0.33±0.01	91.59±0.02	0.71±3.35
RF-R	Raw	0.23±0.01	0.22±0.01	87.86±2.14	52.94±4.99
	1st D	0.09±0.01	0.08±0.01	98.75±0.3	93.91±1.65
	2nd D	0.09±0	0.08±0.01	99.03±0.16	94.55±1.89
	SNV	0.13±0.01	0.13±0.02	96.88±1.05	84.68±4.06
	Smo	0.23±0.01	0.23±0.02	87.96±1.81	51.96±7.17
	Smo+1st D	0.1±0.01	0.09±0.02	98.64±0.26	91.46±3.5
	Smo+2nd D	0.1±0.01	0.09±0.01	98.73±0.2	92.94±1.9
	SNV+Smo	0.13±0.01	0.12±0.01	96.97±0.58	86.7±3.2
	SNV+Smo+1st D	0.09±0.01	0.09±0.02	98.78±0.19	92.59±3.3
	SNV+Smo+2nd D	0.09±0.01	0.08±0.01	98.9±0.14	93.21±1.94
BPNN-R	Raw	0.13±0.02	0.12±0.04	99.44±1.03	85.39±10.04
	1st D	0.09±0	0.09±0.01	100±0	92.39±1.51
	2nd D	0.14±0.01	0.15±0.03	100±0	76.63±12.44
	SNV	0.1±0.02	0.09±0.02	99.86±0.42	92.62±3.02
	Smo	0.12±0.02	0.18±0.26	99.47±0.9	7.03±341.82
	Smo+1st D	0.08±0	0.07±0.01	100±0	94.74±0.88
	Smo+2nd D	0.13±0.01	0.12±0.01	100±0	86.99±2.42
	SNV+Smo	0.08±0.02	0.08±0.02	99.91±0.38	93.63±2.78
	SNV+Smo+1st D	0.08±0	0.07±0.01	100±0	94.73±0.8
	SNV+Smo+2nd D	0.13±0.01	0.13±0.03	100±0	82.67±10.36

All abbreviations are defined in the main text.

Table S5 Output Results of Four Regression Models Based on CARS-IWOA Wavenumber Selection

Models	Propressing	$RMSE_{CV}$	$RMSE_{P}$	R^2_{tra}	R^2_{tes}
PLS-R	Raw	0.09±0	0.1±0.01	93.6±0.69	90.93±1.28
	1st D	0.1±0	0.11±0.01	94.11±0.84	87.87±1.85
	2nd D	0.15±0.01	0.18±0.01	85.06±2.57	70.78±3.63
	SNV	0.09±0	0.1±0.01	94.05±0.44	90.77±1.36
	Smo	0.09±0.01	0.1±0.01	92.99±1.21	90.29±1.41
	Smo+1st D	0.09±0	0.1±0.01	94.75±0.6	90.13±1.37
	Smo+2nd D	0.13±0.01	0.15±0.01	88.69±1.76	77.74±3.39
	SNV+Smo	0.09±0.01	0.1±0.01	93.21±0.99	90.87±1.41
	SNV+Smo+1st D	0.09±0	0.1±0.01	94.56±0.57	90.25±1.15
	SNV+Smo+2nd D	0.13±0.01	0.15±0.01	90.1±1.45	79.58±2.1
SVR	Raw	0.16±0.01	0.15±0.01	93.79±0.45	78.64±3.42
	1st D	0.17±0.02	0.16±0.02	93.26±0.45	75.15±7.5
	2nd D	0.2±0.04	0.2±0.04	93.14±0.66	62.35±16.69
	SNV	0.12±0.01	0.12±0.01	94.42±0.51	86.14±2.57
	Smo	0.16±0.01	0.15±0.02	92.56±2.31	78.59±5.15
	Smo+1st D	0.15±0.01	0.14±0.01	93.42±0.29	80.64±3.63
	Smo+2nd D	0.2±0.04	0.19±0.04	93.05±0.67	63.91±15.23
	SNV+Smo	0.12±0.01	0.12±0.01	94.46±0.53	87.23±2.45
	SNV+Smo+1st D	0.14±0.02	0.13±0.01	93.78±0.49	83.6±2.88
	SNV+Smo+2nd D	0.22±0.04	0.22±0.04	92.81±0.59	55.33±14.65
RF-R	Raw	0.23±0.01	0.22±0.02	87.39±2.42	53.1±6.9
	1st D	0.1±0.01	0.1±0.02	98.4±0.42	91.11±3.21
	2nd D	0.08±0.01	0.07±0.02	99.1±0.33	94.62±2.52
	SNV	0.14±0.01	0.13±0.02	96.54±0.54	83.28±4.37
	Smo	0.23±0.01	0.23±0.02	85.98±2.86	50.73±6.46
	Smo+1st D	0.12±0.01	0.11±0.02	97.67±0.78	88.19±5.52
	Smo+2nd D	0.1±0.01	0.1±0.02	98.53±0.21	90.65±3.21
	SNV+Smo	0.14±0.01	0.14±0.01	96.11±0.77	82.54±3.52
	SNV+Smo+1st D	0.11±0.01	0.11±0.02	97.93±0.79	88.69±5.04
	SNV+Smo+2nd D	0.1±0.01	0.09±0.01	98.69±0.14	92.76±1.96
BPNN-R	Raw	0.14±0.03	0.11±0.03	99.59±0.44	87.31±5.54
	1st D	0.1±0.01	0.1±0.02	100±0	89.84±3.02
	2nd D	0.15±0.02	0.18±0.07	100±0	63.55±41.07
	SNV	0.1±0.01	0.1±0.02	99.84±0.35	90.41±3.79
	Smo	0.12±0.02	0.09±0.02	98.95±0.68	91.08±4.51
	Smo+1st D	0.09±0.01	0.09±0.02	99.99±0	91.54±2.68
	Smo+2nd D	0.13±0.02	0.15±0.03	100±0	78.61±11.95
	SNV+Smo	0.11±0.02	0.12±0.02	99.71±0.46	87.08±5.2
	SNV+Smo+1st D	0.09±0.01	0.1±0.01	100±0	91.29±2.31
	SNV+Smo+2nd D	0.12±0.01	0.13±0.01	100±0	83.5±3.66

All abbreviations are defined in the main text

Figure S1 NIR spectral preprocessing. A, Raw; B, 1st D; C, 2nd D; D, SNV; E, Smo; F, Smo+1st D; G, Smo+2nd D; H, SNV+Smo; I, SNV+Smo+1st D; J, SNV+Smo+2nd D. (All abbreviations are defined in the main text)

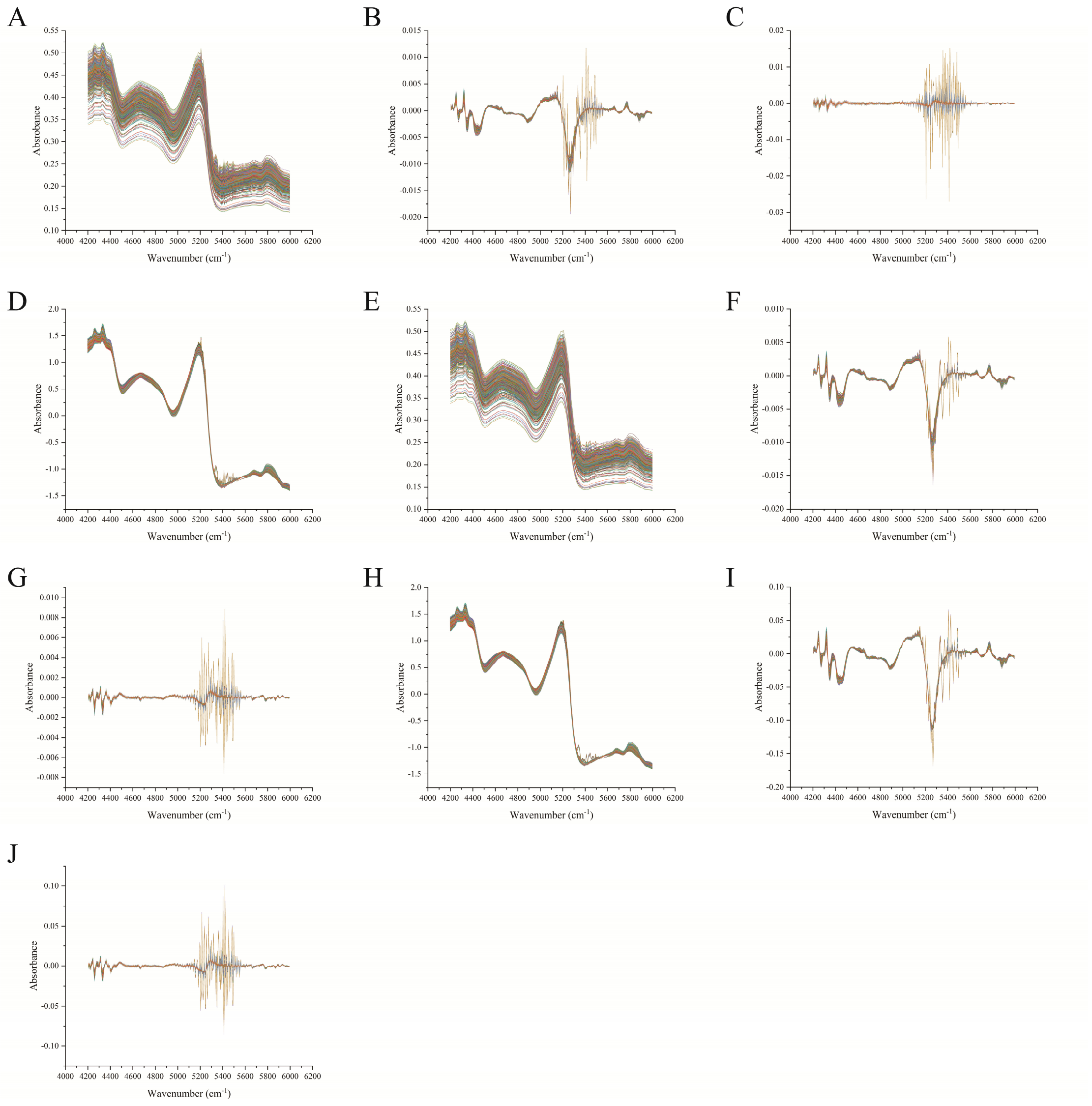

Supplement: Supplementary file 1 [file foods-15-02488-s001.zip › foods-4385411-supplementary.pdf]
